# Racial and Gender Profile of Public Health Faculty in the United States of America

**DOI:** 10.7759/cureus.24998

**Published:** 2022-05-14

**Authors:** Subhash Chander, Sandeep Shelly, Muhammad Haaris Tiwana, Javed Siddiqi, Saleh Fares, Ahmed B Alwazzan, Sarim Faheem, Faisal Khosa

**Affiliations:** 1 Medicine, University at Buffalo, New York, USA; 2 Otolaryngology - Head and Neck Surgery, Emory University School of Medicine, Atlanta, USA; 3 Epidemiology and Biostatistics, Western University, London, CAN; 4 Dentistry, Lahore Medical & Dental College, Lahore, PAK; 5 Neurosurgery, Desert Regional Medical Center, Palm Springs, USA; 6 Neurosurgery, Riverside University Health System Medical Center, Moreno Valley, USA; 7 Neurosurgery, Arrowhead Regional Medical Center, Colton, USA; 8 Neurosurgery, California University of Science and Medicine, Colton, USA; 9 Emergency Medicine, Zayed Military Hospital, Abu Dhabi, ARE; 10 Obstetrics and Gynaecology, King Abdulaziz University College of Medicine, Jeddah, SAU; 11 Science, University of British Columbia Okanagan, Kelowna, CAN; 12 Radiology, Vancouver General Hospital, Vancouver, CAN

**Keywords:** gender-based differences, research productivity, academic rank, public health education, retrospective research

## Abstract

Introduction

In the context of shifting population demographics in the United States (US), a diverse workforce in the discipline of public health can improve outcomes for various populations through the provision of culturally competent public health policies and corresponding research. This study explored the academic, racial, and gender profile of public health faculty in the USA.

Methods

In this retrospective cross-sectional analysis, we analyzed the Association of American Medical Colleges (AAMC) annual report of faculty appointments at US medical schools. Descriptive data analysis was performed for chairperson, full professor, associate professor, assistant professor, instructor, and other positions from 2007 to 2018.

Results

There was a decrease in appointments at all academic ranks from 2007 to 2018 with an absolute change of −239. Overall, most academic positions were occupied by Whites compared to other races, especially in leadership ranks. However, year-by-year analysis showed a gradual decrease in the number of positions held by Whites. Over the last decade, there was a positive trend with a marginally greater number of minorities appointed at academic ranks, specifically Asians. Similarly, no significant change was seen in appointments for Hispanics. Additionally, females occupied a greater number of new positions as compared to their male counterparts except for the higher academic ranks. The data obtained from the AAMC were voluntarily reported and thus may not provide a complete picture of medical faculty in academic medicine.

Conclusion

Women have shown progress in public health faculty positions during our 12-year study period. However, racial and gender incongruity still exists at higher academic ranks and leadership positions. Further research is warranted to explore factors influencing faculty appointment and promotion, and strategies to reduce inequities.

## Introduction

Public health refers to a community-based approach focused on the prevention and management of chronic diseases as well as the promotion of health [1]. The public health workforce is critically important to accomplish the core goals of epidemiology and public health, i.e., assessment, assurance, and policy development [2]. To achieve these goals and to underpin a population health perspective, awareness and understanding of local population needs are required [3]. These include knowledge of social and environmental factors that impact health and illness, assessment and targeting of population needs, the importance of collaboration with community stakeholders, and recognition of international laws [3,4]. These, however, can only be achieved by diverse policymaking and an inclusive research workforce. The United States (US) is culturally diverse as indicated in the 2018 US census, which recorded a total population of 327,167,434, which comprised 5.9% Asian, 18.5% Hispanic, 13.4% African Americans, and 76.3% Whites [5]. Therefore, to serve a diverse US population, there is a need for diversity in the public health workforce. To our knowledge, extensive research has showcased racial and gender disparity among healthcare providers in various medical disciplines [6,7]. However, there are scant data available on the gender and racial composition of the medical public health faculty.

Yu et al.'s study demonstrated that approximately 90% of the leadership positions in academic medicine were retained by Whites [8]. While Caucasians, Asians, and Native Americans were overrepresented by +14.1%, +6.0%, and +1.9%, respectively, in publishing papers [9]. Furthermore, a study assessing the publication productivity showed an underrepresentation of the Hispanic and African American populace in relation to the doctoral degree received and authorship of the research articles [10]. This imbalance, eventually, affects academic advancement and appointment to leadership positions [10].

Nonetheless, racial and gender disparity has been documented in medical and surgical specialties, authorships, funding, editorial boards, and professional societies [11-16]. Our study aimed to explore gender and racial inequality in the academic public health faculty of the US.

## Materials and methods

Institutional review board approval or informed consent was not required due to the use of non-identifiable publicly available data in this study. Our methodology has been validated in several recent publications [17-19]. This retrospective cross-sectional analysis presents data for full-time medical faculty in public health from 2007 to 2018. The data were extracted from the Association of American Medical Colleges (AAMC), which produces an annual report of faculty appointments at medical schools in the US. Race/ethnicity was categorized as White, Asian, Hispanic, African Americans, multiple races, others (American Indians, Alaskan Natives, Native Hawaiian, other Pacific Islands, others), and unknown. Gender was categorized as male and female. Academic rank was categorized as a chairperson, full professor, associate professor, assistant professor, instructor, and other positions. Public health faculty are presently referred to as physicians working in the discipline of public health.

## Results

The total number of academic public health physicians decreased between 2007 and 2018 with an overall absolute change of −239. This was reflected as a decrease in the numbers of all academic positions (chairperson, full professor, associate professor, assistant professor, instructor, and other positions), as shown in Table 1.

**Table 1:** Absolute change in racial representation in all academic groups over 12 years (2007-2018) Others include American Indians, Alaskan Natives, Native Hawaiian, Pacific Islanders, etc. n = 1 physician. "+" denotes increase and "−" denotes decrease.

Academic position and race	2007 (n)	2018 (n)	Absolute change (2018-2007) (n)
Overall academic physicians			
White	743	487	−256
Asian	123	124	1
Black	91	80	−11
Hispanic	25	18	−7
Multiple races	27	26	−1
Unknown	116	150	34
Others	9	10	1
Full professors			
White	203	123	−80
Asian	16	25	9
Black	8	10	2
Hispanic	4	4	0
Multiple races	5	0	−5
Unknown	11	34	23
Others	4	2	−2
Associate professors			
White	163	123	−40
Asian	20	26	6
Black	17	19	2
Hispanic	6	4	−2
Multiple races	2	8	6
Unknown	18	37	19
Others	0	5	5
Assistant professors			
White	255	190	−65
Asian	72	48	−24
Black	49	42	−7
Hispanic	15	9	−6
Multiple races	16	14	−2
Unknown	56	57	1
Others	4	3	−1
Instructors			
White	68	17	−51
Asian	10	5	−5
Black	17	5	−12
Hispanic	0	0	0
Multiple races	0	2	2
Unknown	9	10	1
Others	0	0	0
Other positions			
White	54	34	−20
Asian	5	20	15
Black	0	4	4
Hispanic	0	1	1
Multiple races	4	2	−2
Unknown	22	12	−10
Others	1	0	−1
Chairperson			
White	31	21	−10
Asian	0	3	3
Black	1	2	1
Hispanic	1	0	−1
Multiple races	0	0	0
Unknown	1	3	2
Others	0	0	0

Each year, most of the positions were held by White physicians in all academic physician categories (Table 2). In fact, White race physicians had greater representation across all academic ranks followed by Asians, Blacks, and Hispanics, respectively. On average, Asians demonstrated prodigious growth at every academic rank across the 12-year study period. This showed encouraging results for Asian ethnicity. For instance, the data affirm that Asians comprised 14% of all academic physician positions, 13% of full professors, assistant professors, and instructor positions, and 12% of associate professor positions during the year 2018. An increase of 3%, 7%, 7%, and 3% was seen in respective ranks over the 12-year study period. One notable exception was for the rank of assistant professor, where data showed a 2% decrease. There was an appreciable growth in the chairperson position(s) for two groups; Asians accounted for 0% in 2007 vs. 10% in 2018 and African Americans accounted for 3% in 2007 vs. 7% in 2018.

**Table 2:** Yearly breakdown of various academic groups over 12 years (2007-2018) by race and gender Others include American Indians, Alaskan Natives, Native Hawaiian, Pacific Islanders, etc. n = 1 physician. "+" denotes increase and "−" denotes decrease.

Academic position and race	2007 (n)	2008 (n)	2009 (n)	2010 (n)	2011 (n)	2012 (n)	2013 (n)	2014 (n)	2015 (n)	2016 (n)	2017 (n)	2018 (n)	Absolute change (2018-2007) (n)	Relative change (%)
All academic physicians														
Race														
White	743	679	631	609	593	524	527	496	507	472	470	487	−256	−34.45
Asian	123	118	118	124	126	121	119	114	118	122	125	124	1	0.81
Black	91	81	77	79	85	81	74	80	81	81	80	80	−11	−12.09
Hispanic	25	22	23	23	23	17	17	15	18	18	18	18	−7	−28.00
Multiple races	27	26	29	30	28	24	22	21	24	30	31	26	−1	−3.70
Unknown	116	137	146	156	146	140	129	132	129	141	150	150	34	29.31
Others	9	6	6	8	9	8	7	5	5	7	9	10	1	11.11
Gender														
Male	561	519	504	496	479	438	426	398	406	396	394	407	−154	−27.45
Female	573	550	526	533	531	477	469	465	476	475	489	488	−85	−14.83
Full professors														
Race														
White	203	186	176	173	171	154	146	134	132	116	115	123	−80	−39.41
Asian	16	15	16	16	19	18	19	19	19	19	25	25	9	56.25
Black	8	7	6	5	6	6	6	8	7	9	8	10	2	25.00
Hispanic	4	4	5	4	5	3	5	3	2	2	3	4	0	0.00
Multiple races	5	4	4	4	4	3	1	1	0	0	0	0	−5	−100.00
Unknown	11	13	15	15	16	18	20	20	25	27	30	34	23	209.09
Others	4	2	2	2	2	2	2	1	1	1	1	2	−2	−50.00
Gender														
Male	175	158	150	147	144	126	119	107	107	94	95	100	−75	−42.86
Female	76	73	74	72	79	78	80	79	79	80	87	98	22	28.95
Associate professors														
Race														
White	163	151	150	145	150	129	140	125	130	118	121	123	−40	−24.54
Asian	20	21	20	20	30	30	27	24	24	28	22	26	6	30.00
Black	17	17	17	18	17	18	16	14	19	18	19	19	2	11.76
Hispanic	6	6	7	8	7	4	1	1	3	3	5	4	−2	−33.33
Multiple races	2	2	3	2	1	1	3	4	4	6	7	8	6	300.00
Unknown	18	19	21	26	24	25	30	28	32	33	34	37	19	105.56
Others	0	0	0	1	1	1	1	2	2	4	5	5	5	500.00
Gender														
Male	123	112	110	99	98	91	97	89	97	104	101	112	−11	−8.94
Female	103	104	108	121	132	117	121	109	117	106	112	110	7	6.80
Assistant professors														
Race														
White	255	226	213	194	186	176	173	171	182	185	185	190	−65	−25.49
Asian	72	69	72	76	65	63	58	54	50	50	55	48	−24	−33.33
Black	49	43	42	37	37	32	29	35	39	43	42	42	−7	−14.29
Hispanic	15	12	11	10	11	9	9	9	11	11	8	9	−6	−40.00
Multiple races	16	16	19	21	21	18	15	13	15	18	18	14	−2	−12.50
Unknown	56	61	62	66	60	58	50	50	54	62	63	57	1	1.79
Others	4	3	3	3	4	4	3	1	1	2	3	3	−1	−25.00
Gender														
Male	198	178	175	177	174	175	155	146	155	154	160	155	−43	−21.72
Female	269	252	247	230	210	185	182	187	197	217	214	208	−61	−22.68
Instructors														
Race														
White	68	62	45	48	43	33	30	23	23	17	18	17	−51	−75.00
Asian	10	9	7	6	3	2	6	4	4	2	4	5	−5	−50.00
Black	17	14	12	19	23	24	19	17	13	8	7	5	−12	−70.59
Hispanic	0	0	0	1	0	0	0	0	0	0	0	0	0	0.00
Multiple races	0	0	0	0	0	0	1	2	3	4	4	2	2	200.00
Unknown	9	13	16	12	12	13	9	9	10	9	9	10	1	11.11
Others	0	0	0	1	1	1	1	1	1	0	0	0	0	0.00
Gender														
Male	36	38	32	34	27	23	22	17	16	12	10	9	−27	−75.00
Female	68	60	48	53	55	50	44	39	38	28	32	30	−38	−55.88
Other positions														
Race														
White	54	54	47	49	43	32	38	43	40	36	31	34	−20	−37.04
Asian	5	4	3	6	9	8	9	13	21	23	19	20	15	300.00
Black	0	0	0	0	2	1	4	6	3	3	4	4	4	400.00
Hispanic	0	0	0	0	0	1	2	2	2	2	2	1	1	100.00
Multiple races	4	4	3	4	2	2	2	1	2	2	2	2	−2	−50.00
Unknown	22	31	32	37	34	26	20	25	8	10	14	12	−10	−45.45
Others	1	1	1	1	1	0	0	0	0	0	0	0	−1	−100.00
Gender														
Male	29	33	37	39	36	28	33	39	31	32	28	31	2	6.90
Female	57	61	49	57	55	42	42	51	45	44	44	42	−15	−26.32
Chairperson														
Race														
White	31	29	26	26	25	25	23	21	23	21	20	21	−10	−32.26
Asian	0	0	0	0	1	1	1	1	3	3	3	3	3	300.00
Black	1	1	2	2	2	2	2	2	2	2	2	2	1	100.00
Hispanic	1	1	1	1	1	0	0	0	0	0	0	0	−1	−100.00
Multiple races	0	0	0	0	0	0	0	0	0	0	1	0	0	0.00
Unknown	1	1	1	1	1	1	2	2	3	3	3	3	2	200.00
Others	0	0	0	0	0	0	0	0	0	0	0	0	0	0.00
Gender														
Male	30	27	23	22	21	20	19	18	21	20	19	20	−10	−33.33
Female	4	5	7	8	9	9	9	8	10	9	10	9	5	125.00

**Figure 1:**
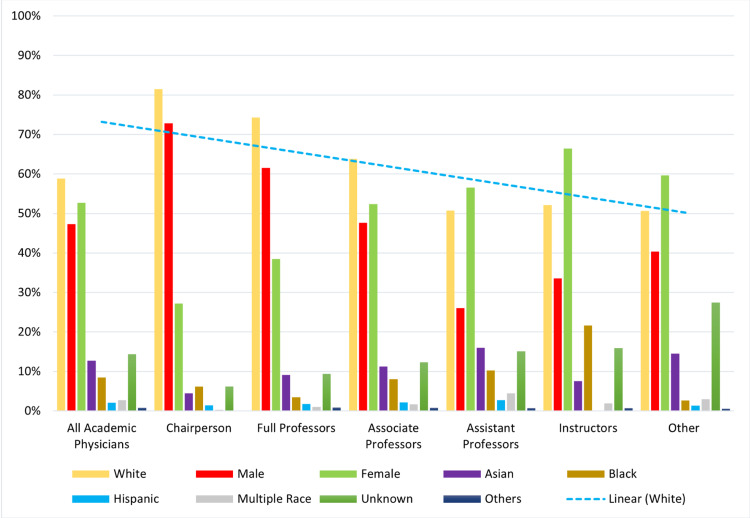
Average racial distribution of new academic positions offered between 2007 and 2018 Others include American Indians, Alaskan Natives, Native Hawaiian, Pacific Islanders, etc.

White race physicians held most academic positions during the 12-year study period. A year-by-year analysis demonstrated that there was a reduction in the percentage of White faculty members in many of the positions (Table 2). For example, an absolute change of −12%, −3%, and −21% was seen in academic physicians, assistant professors, and instructor positions, respectively. Furthermore, an absolute change of −19% was reported across full professor, associate professor, and chairperson ranks for white race physicians. The representation of Hispanics did not increase and instead showed a decrease in their appointment for most of the academic ranks during the 12-year study period (Figure 1).

Among the junior ranks (assistant professor, instructor, and other positions), females occupied a greater number of new positions in comparison to their male counterparts (Table 3). For instance, the chairperson data show an absolute change of +230 for males compared to +93 for females. Similarly, an absolute change of +1347 was found for males vs. +879 for females for full professors over the 12-year study period (Table 2). However, analyzing the data considering only the female gender showed a significant linear growth; chairperson ranks increased from 12% in 2007 to 31% in 2018, and full professor rank increased from 30% in 2007 to 49% in 2018. There was an absolute change of −19% in the number of leadership positions held by men and an equal proportion of the increase in the number occupied by females. A similar trend was seen for the full professor and associate professor positions. This indicates that females rather than males replaced most positions.

**Table 3:** Absolute change in gender representation in all academic groups over 12 years (2007-2018) "+" denotes increase and "−" denotes decrease.

All academic levels in public health		2007 (n)	2018 (n)	Absolute change (n)
All academic levels	Male	561	407	−154
	Female	573	488	−85
Chairperson	Male	30	20	−10
	Female	4	9	+5
Full professors	Male	175	100	−75
	Female	76	98	+22
Associate professors	Male	123	112	−11
	Female	103	110	+7
Assistant professors	Male	198	155	−43
	Female	269	208	−61
Instructors	Male	36	9	−27
	Female	68	30	−38
Other positions	Male	29	28	−1
	Female	57	44	−3

## Discussion

The results of this study provide pilot data for planned large-scale future investigations in public health. Our study highlighted that African Americans and Hispanics were minimally represented in higher academic positions from 2007 to 2018, as compared to Whites and Asians. Moreover, Hispanics were under-represented in nearly all academic positions. In 2007, Whites held the majority of academic positions, i.e., chairperson (91%), full professors (81%), associate professors (72%), assistant professors (55%), and instructors (65%). However, the year-by-year analysis presented a decrease in the number of positions held by Whites in almost all positions. Apart from this, fewer academic positions were occupied by females in high-ranking leadership positions (chairperson, full professor, associate professor). Thus, presenting a clear picture of the under-representation of the female gender in higher academic ranks.

Our analysis is consistent with previous studies that characterized the gender, racial, and ethnic distribution of academic public health physician ranks [20-22]. While maintaining their dominance across all positions, our study showed a decrease in the overall number of positions held by Whites in each position. It also showed a simultaneous increase in the positions held by Asians. This finding coincides with Lee et al.'s study [21] describing a decreasing proportion of White faculty members from total academic physicians and a concurrent increasing number of the Asian faculty within the US academic workforce. Furthermore, recent studies have shown that disparity in academia is multifactorial, including biases in hiring, promotion, and compensation [20,22,23], all of which may limit underrepresented minorities in medicine (URiM) faculty recruitment, promotion, and retention.

Interestingly, our study showed promising results for Asians, particularly in higher academic ranks and leadership positions. There were no Asian chairpersons reported in 2007 but this proportion increased to 10% in 2018. Probably, this increase is due to the simultaneous growth in Asian immigrants having international medical degrees that add to a greater number of Asian faculty members. A study done by Fang et al. also reported a similar trend with the rise of Asian faculty in academic medicine and asserted that this is due to an increase in the general Asian population of the USA [24]. This may also be due to US immigration trends. Currently, more than one million people immigrate to the US and amongst new immigrant arrivals, Asians have largely outnumbered Hispanics since 2009 [25]. It is worth noticing that there has been no change in the representation of Hispanics in public health academic faculty. This trend shows that Asians and Whites are overrepresented in the academic discipline.

Considering gender, the data showed an increase in the number of females from 2007 to 2018. However, as compared to males, there remains a gap in the female representation in leadership positions (2007: 88% vs. 12%; 2018: 69% vs. 31%). These findings are consistent with Bickel's study [26] done in 2000, where women consisted of only 11% of the full professors as compared to 31% of males. This highlighted the lack of statistical improvement in gender balance within academia since this prior study.

Institutional culture plays a crucial role in promoting women in science and medicine [26]. Several publications have documented a persistent and damaging culture of behavior that limits the participation of women in academics [22,23,27]. URiM involvement in research enhances minority recruitment into research studies and can help increase participation from traditionally hard-to-reach populations. Some studies also support the basis that diverse institutions train physicians who more effectively serve minority communities. Hence, improving representation can indirectly improve health outcomes in minorities by understanding community needs, aspirations, and culture [13,28-30]. Further research is needed to explore policies for recruitment and promotion that may be contributing to racial and gender disparity in public health academic medicine and the ways to rectify the gaps.

Strengths and limitations

Our study has its share of strengths and limitations. The major strength is the utilization of a national data repository over a 12-year period. Additionally, this study buttresses the findings of various previous publications and provides knowledge about the positive trend toward female empowerment. This study also has its share of limitations. First, the AAMC faculty appointment is voluntarily reported and may not include all medical faculty in academic medicine. Also, evaluating percentage reporting was out of the scope of this study. Similarly, the study did not determine the percentage of the job applications for various positions. Furthermore, the promotion process is highly variable within and between institutions, which may affect the internal validity of the data. Another important limitation is the non-availability of the non-binary gender community in the data. Future studies should be directed to fill this knowledge gap. The retrospective cross-sectional design of this study as compared to a longitudinal design limits the power of the results but provides a better representation of the current years.

## Conclusions

Despite an upward trend of 19% since 2007 toward the representation of women and minorities in faculty positions, 69% of positions are still predominantly held by White race male physicians. Continued support and retention of underrepresented minorities are pivotal to further improving their future representation in academic medicine.
